# Association of osteoprotegerin with impaired glucose regulation and microalbuminuria: the REACTION study

**DOI:** 10.1186/s12902-015-0067-5

**Published:** 2015-12-01

**Authors:** Yixin Niu, Zhen Yang, Xiaoyong Li, Weiwei Zhang, Shuai Lu, Hongmei Zhang, Xueru Chen, Lingfei Zhu, Yin Xing, Guang Ning, Li Qin, Qing Su

**Affiliations:** Department of Endocrinology, Xinhua Hospital, Shanghai Jiaotong University School of Medicine, 1665 Kongjiang Road, Shanghai, 200092 China; Department of Endocrinology, Xinhua Hospital Chongming Branch, Shanghai Jiaotong University School of Medicine, Shanghai, China; Shanghai Institute of Endocrinology and Metabolism, Department of Endocrine and Metabolic Diseases, Shanghai Clinical Center for Endocrine and Metabolic Diseases, Ruijin Hospital, Shanghai Jiao Tong University School of Medicine, Shanghai, China; The Key Laboratory of Endocrine Tumors and the Division of Endocrine and Metabolic Diseases, E-Institute of Shanghai Universities, Shanghai, China

**Keywords:** Impaired glucose regulation, Osteoprotegerin, Microalbuminuria

## Abstract

**Background:**

High osteoprotegerin (OPG) has been reported in association with insulin resistance and type 2 diabetes. We aimed to evaluate the association of serum OPG with impaired glucose regulation (IGR) and microalbuminuria among middle-aged and older Chinese.

**Methods:**

Serum OPG was measured in 599 individuals with normal glucose regulation, 730 with impaired glucose regulation and 327 newly diagnosed patients with diabetes. Serum OPG was measured using ELISA methods and urine albumin/creatinine ratio was used to determine the urinary albumin excretion.

**Results:**

Serum OPG levels were significantly higher in subjects with isolated impaired fasting glucose, isolated impaired glucose tolerance, combined impaired fasting glucose/impaired glucose tolerance and diabetes than in those with normal glucose regulation, whereas serum OPG levels were not different in the four groups with dysregulation of glucose metabolism. OPG was associated with a higher risk for IGR (OR 1.108 for each 0.1 μg/l increase in OPG, 95 % CI 1.009–1.117, *p* = 0.01) after adjustment for gender, age, BMI, current smoking and alcohol intake, family history of diabetes, homeostasis model assessment of insulin resistance (HOMA-IR), lipid profile; the corresponding OR of combined impaired glucose regulation and type 2 diabetes was 1.121 (95 % CI 1.101–1.141, *p* = 0.0005). OPG was associated with the risk of microalbuminuria (OR 1.025, 95 % CI 1.006–1.044, *p* = 0.02) after adjustment for gender, age, current smoking, current alcohol intake, family history of diabetes, BMI, waist/hip ratio, HOMA-IR, eGFR and lipid profile.

**Conclusions:**

Serum OPG level is closely and independently associated with IGR and is an independent risk factor for microalbuminuria.

## What’s new?

In this study, we found a strong association between serum OPG levels and the risk of impaired glucose regulation in a large Chinese cohort aged 40 years or older.

Serum OPG was also associated with a higher risk of microalbuminuria independent of various confounding factors.

To our knowledge, this is the first study to evaluate the relationship between OPG levels and impaired glucose regulation in a large-scale population.

## Background

Osteoprotegerin (OPG), a soluble member of the tumor necrosis factor (TNF) receptor superfamily is a decoy receptor for the receptor activator of nucleus factor-κB ligand (RANKL) and TNF-related apoptosis-inducing ligand (TRAIL) [1, 2], has been identified as having an effect on systemic insulin sensitivity and glucose homeostasis [3–5]. The association of serum OPG with insulin resistance has been most commonly studied; however, the results from these studies are not consistent. While serum OPG levels were increased in certain metabolic disorders, such as obesity, type 2 diabetes, and polycystic ovarian syndrome, that were closely related to insulin resistance [3–11], some other studies reported that serum OPG levels was not related to insulin resistance or even negative related to insulin resistance [12, 13].

Insulin resistance is a conspicuous characteristic of prediabetic states [14]. Impaired glucose regulation (IGR), also termed as prediabetes consisting of impaired fasting glucose and/or impaired glucose tolerance (IFG and/or IGT) is definitely a risk factor for type 2 diabetes and cardiovascular disease [15]. Recently, epidemiological studies showed that the prediabetes is abruptly increased in the world, as well as in China [16]. Elevated serum OPG levels have been reported in connection with IGT in study with small sample sizes [17]; however, the association of OPG with isolated IFG, isolated IGT and combined IFG and IGT has rarely been studied. Thus, it is indispensable to evaluate the association of serum OPG with type 2 diabetes, as well as with prediabetes. Microalbuminuria, which is an early indicator of renal dysfunction, represents a risk factor for atherosclerosis and cardiovascular disease independent of conventional risk factors [18]. Previous studies demonstrated that microalbuminuria was associated with insulin resistance in patients with type 2 diabetes as well as in patients without diabetes [19]. Studies have also shown an association of serum OPG with albumin excretion in type 2 diabetes, although this relationship was not fully elucidated [7].

In the present study, we evaluated the association of serum OPG with states of glucose metabolism and urinary albumin excretion in a cross-sectional population study of middle-aged and older Chinese subjects.

## Methods

### Study population

In 2011 China a national survey of Risk Evaluation of cancers in Chinese diabetic Individuals: a longitudinal (REACTION) study, which was conducted among 259,657 adults, aged 40 years and older in 25 communities across mainland China, from 2011 to 2012 [20]. The data presented in this article are based on the baseline survey of subsamples from Shanghai in eastern China. All studied individuals came from the Chongming District in Shanghai, China. Informed consent was obtained from all participants, and approval was given by the Institutional Review Board of Xinhua Hospital affiliated with Shanghai Jiaotong University School of Medicine. The study population, design and protocols of this cohort study have been described previously [21]. Briefly, a two-stage stratified sampling method was used. First, 12 residential communities or streets were randomly selected from the Chongming District. Of these, 8 urban communities and 4 rural communities were chosen to represent people with high to low socioeconomic status. Secondly, Within each community/street, all eligible individuals were sampled, with the exception that in households with more than one eligible individual, one individual was randomly selected. During the recruiting phase, inhabitants aged ≥40 years in these 12 communities were invited by telephone or door-to-door visit to participate in this study.

A total of 9,930 subjects were recruited and agreed to participate in the first step of our survey. After the exclusion of 990 individuals with self-reported diabetes, a random subcohort of 1800 individuals was selected from all participants of the study population. Although we considered missing anthropometry as exclusion criteria, none of the subcohort members qualified for exclusion here. Furthermore, we excluded 123 participants for whom not all biomarkers were available and 21 individuals with either unexplainable low plasma glucose concentrations (<2.8 mmol/l). Altogether, 1,656 participants were included in the random subcohort for analyses. The process of randomly selecting a subcohort, together with the appropriate statistics for this type of research design, renders the results generalizable without measurements of biomarkers in the entire cohort. [22] A comparison of the randomly selected subcohort, before and after exclusions, and the full cohort was performed. There were no significant differences in age, BMI, or sex distribution between full and subcohort before exclusions, suggesting that the subcohort was representative for the full cohort.

### Data collection

A standardized questionnaire was used by trained physicians to collect information such as age, gender, smoking (yes/no), alcohol drinking (yes/no). Physical activity level was classified as low, moderate, or high according to the International Physical Activity Questionnaire scoring protocol. According to participants’ responses to the corresponding questions, family history of diabetes was classified as yes or no.

The details of anthropometric measurements including height, weight, waist circumference, hip circumference were carried by trained physicians. Blood pressure was measured at the right arm with an automated electronic device (OMRON Model1 Plus; Omron Company, Kyoto, Japan) three times consecutively with 1 min intervals after at least 5 min rest in the seated position; the three readings were averaged for analysis. Body mass index (BMI) was calculated as weight in kilograms divided by the square of height in meters.

All subjects were assessed after overnight fasting for at least 10 h. Overnight fasting and 2 h OGTT blood samples were collected in tubes containing EDTA and were centrifuged at 4 °C and stored at −80 °C until analysis. The fasting glucose, glucose 2 h after oral glucose tolerance test, total cholesterol (TC), triglycerides, low-density lipoprotein (LDL) cholesterol and high-density lipoprotein (HDL) cholesterol were measured on an automatic analyzer (Hitachi 7080; Tokyo, Japan). Fasting insulin was determined by RIA (Linco Research, St. Charles, MO). The homeostasis model assessment of insulin resistance (HOMA-IR) was calculated according to the equation described by Matthews et al. [23] The abbreviated Modification of Diet in Renal Disease formula recalibrated for Chinese was used to estimate glomerular filtration rate expressed in milliliters per minute per 1.73 m^2^: estimated glomerular filtration rate (eGFR) = 186 × [serum creatinine × 0.011]^-1.154^ × [age]^-0.203^ × [0.742 if female] × 1.233, where serum creatinine is expressed as micromoles per liter and 1.233 is the adjusting coefficient for Chinese [24].

Urinary albumin and creatinine concentrations were determined using the first-void sterile urine sample in the early morning by rate nephelometry (Beckman Coulter, Fullerton, CA, USA) and alkaline nitroxanthic acid method, respectively. The sensitivity of the urinary albumin assay was 2.0 g/l. The albumin/creatinine ratio (ACR) was calculated as milligrams of urinary albumin excretion per gram of urinary creatinine and used for the diagnosis of normal, micro- and macroalbuminuria, defined as an ACR <3.4, 3.4–34, and >34 mg/mmol, respectively.

### Measurements of serum osteoprotegerin

The serum OPG was determined in duplicate by ELISA with Duoset kit (DY805; R&D Systems, Minneapolis, MN) as recommended by the manufacturer. The ELISA system had an intraassay coefficient of variation of 3–9 % and an interassay coefficient of variation of 3–10 %, respectively.

### Definitions

Impaired glucose regulation was defined as impaired fasting glucose (fasting plasma glucose level ≥6.1 and <7.0 mmol/l) and/or impaired glucose tolerance (IGT, 2 h OGTT plasma glucose level ≥7.8 and <11.1 mmol/l). Isolated IFG: fasting plasma glucose ≥6.1 mmol/l and <7.0 mmol/l and 2 h OGTT plasma glucose <7.8 mmol/l. Isolated IGT: 2 h OGTT plasma glucose ≥7.8 mmol/l and <11.1 mmol/l and fasting glucose <6.1 mmol/l. IFG/IGT: fasting plasma glucose 6.1 mmol/l to 6.9 mmol/l and 2 h OGTT plasma glucose 7.8 mmol/l to 11.0 mmol/l. Type 2 diabetes was diagnosed according to the 1999 World Health Organization criteria (fasting plasma glucose level ≥7.0 mmol/l and/or 2 h OGTT plasma glucose level ≥11.1 mmol/l). A fasting glucose level lower than 6.1 mmol/l and 2 h OGTT plasma glucose level below 7.8 mmol/l were defined as normal glucose regulation (NGR).

### Statistical analysis

Normally distributed data were expressed as means ± SD, whereas variables with a skewed distribution were reported as median (interquartile range) and log transformed to approximate normality before analysis. Comparisons of means and proportions were performed with the standard normal z and *χ*2 tests, respectively. Homogeneity of groups was determined when the means showed significant differences. Means of these groups were compared by the Student–Newman–Keuls method.

To allow for covariates and confounders, we performed analysis of covariance and multiple linear and logistic regressions. Univariate and multivariable stepwise logistic regression analysis were used to investigate the association of serum OPG with clinical and biochemical characteristics. The forward regression procedure was used to obtain determinants of serum OPG, and we considered as potential covariates age, sex, body mass index, waist circumference, fasting plasma glucose, 2 h OGTT plasma glucose, log10 fasting serum insulin, log10 HOMA-IR index, serum concentrations of triacylglycerols, total cholesterol, HDL- and LDL-cholesterol, systolic and diastolic blood pressure. To study the association of impaired glucose regulation with OPG, we defined participants with normal glucose regulation as 0 (*n* = 599) and impaired glucose regulation as 1 (*n* = 730), and excluded patients with type 2 diabetes from the logistic regression analyses. For the association of combined impaired glucose regulation and type 2 diabetes with OPG, we defined participants with normal glucose regulation as 0 (*n* = 599) and combined impaired glucose regulation and type 2 diabetes as 1 (*n* = 1,057) in the logistic regression analyses. To investigate the association of microalbuminuria with OPG, we defined participants with normal urinary albumin excretion as 0 (*n* = 1,539) and microalbuminuria as 1 (*n* = 103), excluding macroalbuminuria (*n* = 14) from the logistic regression analyses. For association of combined micro- and macroalbuminuria with OPG, we defined participants with normal urinary albumin excretion as 0 (*n* = 1,539) and combined micro- and macroalbuminuria as 1 (*n* = 117). Data management and statistical analysis were performed with the SPSS Statistical Package (version 13.0; SPSS Inc., Chicago, IL).*P* values < 0.05 were considered statistically significant.

## Results

### Characteristics of the participants

The study involved 599 participants with NGR, 730 with IGR and 327 with newly diagnosed type 2 diabetes (Table 1). Among the participants with IGR, 230 (13.9 %) had isolated IFG, 286 (17.3 %) had isolated IGT, and 214 (12.9 %) had combined IFG/IGT. There was no significant difference in current smoking and alcohol intake between these groups (both *p* > 0.05). The NGR and diabetes groups had, respectively, the most favourable and unfavourable metabolic profiles. There was no significant difference in age, BMI, waist/hip ratio, fasting serum insulin, HDL-cholesterol, white blood cell count, postmenopausal women between the groups with isolated IFG, isolated IGT and combined IFG/IGT. The isolated IGT and combined IFG/IGT groups had higher 2 h OGTT plasma glucose than the isolated IFG and NGR groups. The isolated IGT and combined IFG/IGT groups had higher serum fasting insulin concentration and urinary ACR than the isolated IFG and NGR groups. The metabolic profile of the combined IFG/IGT group showed a trend towards similarity to the type 2 diabetes group.

**Table 1:** Characteristics of the study population

Characteristic	NGR (0)	IGR			Type 2 diabetes (4)	*p* value	Homogeneity of groups
		Isolated IFG (1)	Isolated IGT (2)	IFG and IGT (3)			
n	599	230	286	214	327	-	-
Male, n(%)	200 (43.4)	96 (41.7)	84 (29.4)	86 (40.2)	129 (39.4)	0.008	-
Age (years)	54.2 ± 8.3	56.3 ± 7.5	55.6 ± 8.1	56.9 ± 7.4	57.3 ± 7.7	<0.0001	(0) (1,2,3,4)
BMI (kg/m^2^)	24.4 ± 3.7	25.1 ± 3.4	24.7 ± 3.4	25.1 ± 3.9	25.5 ± 3.6	<0.0001	(0,1,2,3) (1,3,4)
Waist circumference (cm)	84.0 ± 10.3	86.8 ± 10.0	84.5 ± 9.7	85.3 ± 11.8	88.0 ± 10.8	<0.0001	(0,2,3) (1.3) (1,4)
Waist/hip ratio	0.88 ± 0.08	0.90 ± 0.09	0.89 ± 0.07	0.89 ± 0.13	0.93 ± 0.16	0.012	(0,1,2,3) (1,2,3,4)
Fasting plasma glucose (mmol/l)	5.5 ± 0.3	6.4 ± 0.2	5.6 ± 0.3	6.4 ± 0.2	7.5 ± 2.3	<0.0001	(0,2) (1,3) (4)
2 h OGTT plasma glucose (mmol/l)	6.1 ± 1.1	6.3 ± 1.0	9.0 ± 0.9	9.1 ± 0.9	13.2 ± 5.0	<0.0001	(0,1) (2,3) (4)
Fasting serum insulin (mmol/l)	5.50 (3.90–7.63)	6.20 (4.30–8.78)	6.25 (4.23–8.60)	6.80 (4.50–9.90)	7.20 (4.70–10.20)	<0.0001	(0) (1,2,3) (4)
Hemoglobin A1c (%)	5.4 ± 0.3	5.6 ± 0.4	5.5 ± 0.3	5.7 ± 0.4	6.3 ± 1.4	<0.0001	(0) (1,2) (1,3) (4)
HOMA-IR	1.39 (0.95–1.87)	1.79 (1.26–2.53)	1.64 (1.15–2.31)	1.93 (1.33–2.80)	2.37 (1.47–3.39)	<0.0001	(0) (1,2) (1,3) (4)
Systolic blood pressure (mmHg)	130 ± 19	136 ± 20	132 ± 19	139 ± 17	141 ± 20	<0.0001	(0,2) (1) (3,4)
Diastolic blood pressure (mmHg)	81 ± 11	82 ± 10	82 ± 10	84 ± 11	84 ± 11	<0.0001	(0,1,2) (1,3) (3,4)
Total cholesterol (mmol/l)	4.81 ± 0.83	5.09 ± 0.84	4.92 ± 0.86	5.13 ± 0.91	5.23 ± 0.91	<0.0001	(0,2) (1,3,4)
Triacylglycerol (mmol/l)	1.39 ± 0.85	1.75 ± 1.36	1.83 ± 1.11	2.20 ± 1.85	2.17 ± 1.55	<0.0001	(0) (1,2) (3,4)
LDL-cholesterol (mmol/l)	2.67 ± 0.70	2.83 ± 0.68	2.68 ± 0.68	2.81 ± 0.68	2.87 ± 0.77	<0.0001	(0,2) (1,3,4)
HDL-cholesterol (mmol/l)	1.37 ± 0.30	1.34 ± 0.30	1.32 ± 0.29	1.28 ± 0.35	1.32 ± 0.33	0.009	(0,1,2,4) (1,2,3,4)
White blood cell count (×109)	5.7 ± 1.3	6.2 ± 1.5	6.2 ± 1.5	6.4 ± 1.6	6.5 ± 1.7	<0.0001	(0) (1,2,3) (3,4)
ACR (mg/g)	7 (4–11)	8 (5–14)	8 (4–13)	8 (5–15)	10 (6–20)	<0.0001	(0) (1,2,3) (4)
Serum creatinine concentration (mmol/l)	62.0 (56.4–69.1)	66.1 (60.4–74.3)	62.5 (57.4–69.4)	65.6 (59.4–73.0)	66.3 (60.2–73.9)	0.55	(0,1,2,3,4)
GFR (ml min^−1^ 1.73 m^−2^)	126.7 (112.7–142.7)	120.6 (108.4–136.3)	122.7 (110.3–138.9)	120.4 (106.8–133.8)	118.5 (105.6–134.9)	0.029	(0,1,2,3) (4)
Current smoking, n (%)	147 (24.5)	56 (24.3)	59 (20.6)	65 (30.4)	78 (23.9)	0.172	-
Alcohol intake, n (%)	148 (24.7)	68 (30.0)	72 (25.2)	72 (33.6)	93 (28.4)	0.099	-
Postmenopausal women, n (%)	301 (50.3)	102 (44.3)	153 (53.5)	101 (47.2)	156 (47.7)	0.149	-
OPG (μg/l)	2.63 ± 0.95	2.95 ± 1.01	2.98 ± 1.03	2.96 ± 1.00	3.02 ± 1.09	<0.0001	(0) (1,2,3,4)

Values are mean ± SD or median (interquartile range) or number (proportion)

*p* values were for the ANOVA or *χ*2 analyses across the five groups

### Serum OPG levels by sex and age

There was no significant difference in serum OPG levels between the men and women (p > 0.05). Age was positively associated with serum OPG levels (r = 0.377, *p* < 0.0001). OPG concentrations increased by 0.30 μg/ml with each 10 year increase in age (*p* < 0.0001).

### Determinants of serum OPG levels

Age, 2 h OGTT plasma glucose, HbA1c, log10 HOMA-IR, triacylglycerol and log10-ACR were independent determinants for serum OPG concentrations in a stepwise linear regression analysis (Table 2). Furthermore, serum OPG levels were associated with white blood cell count (r = 0.225, *p* < 0.0001) in this sudy.

**Table 2:** Univariate and stepwise regression analysis with serum OPG concentration as a dependent variable

Co-variable	Univariate		Stepwise	
	r	*p* value	Beta	*p* value
Age (years)	0.377	<0.0001	0.331	<0.0001
Sex (male = 1, female = 2)	–0.12	0.440	-	-
Body mass index (kg/m2)	0.068	0.193	-	-
Waist/hip ratio	0.023	0.262	-	-
Fasting plasma glucose (mmol/l)	0.191	0.007	-	-
2 h OGTT plasma glucose (mmol/l)	0.266	<0.0001	0.222	0.002
Log_10_ fasting serum insulin (mmol/l)	–0.125	0.056	–0.175	0.021
Hemoglobin A1c (%)	0.251	0.001	-	-
Log_10_ HOMA-IR	0. 29	<0.0001	0.20	0.003
Systolic blood pressure (mmHg)	0.104	0.092	-	-
Diastolic blood pressure (mmHg)	0.003	0.483	-	-
Total cholesterol (mmol/l)	0.113	0.075	-	-
Triacylglycerol (mmol/l)	0.222	0.002	0.255	0.001
LDL-cholesterol (mmol/l)	0.045	0.282	-	-
HDL-cholesterol (mmol/l)	–0.057	0.235	-	-
Log_10_ ACR	0.09	0.05	0.16	0.025
Blood cell count	0.225	<0.0001	-	-

Beta, Regression coefficient; r, Pearson correlation coefficient

### OPG in impaired glucose regulation and newly diagnosed type 2 diabetes

**Fig. 1:**
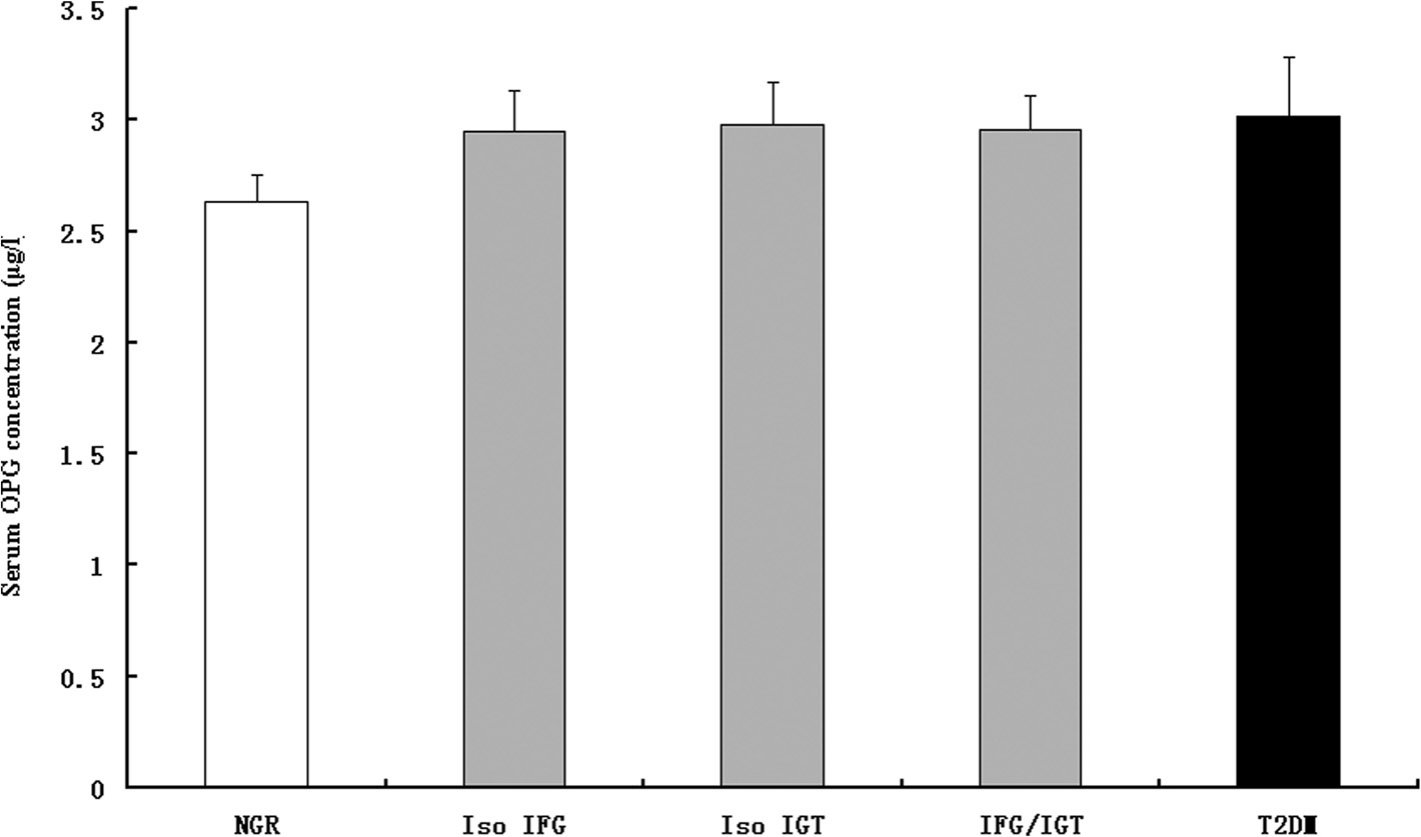
Adjusted means (±SEM) of serum OPG concentrations in subjects with NGR, IGR (isolated IFG [Iso IFG], isolated IGT [Iso IGT] and IFG/IGT) and type 2 diabetes mellitus (T2DM). The covariables included age, sex, BMI, current smoking, current alcohol intake, menopausal status and the family history of diabetes. Subjects with type 2 diabetes mellitus and IGR (isolated IFG, isolated IGT and IFG/IGT) had higher concentrations of OPG than those with NGR (both *p* < 0.0001). There was no significant difference among the subgroups of IGR (*p* = 0.35) and between the IGR and type 2 diabetes mellitus groups (*p* = 0.61)

Serum OPG concentrations were significantly higher in subjects with isolated IFG, isolated IGT, combined IFG and IGT, and newly diagnosed type 2 diabetes compared with concentrations in participants with NGR (2.95, 2.98, 2.96, 3.02 μg/l vs 2.63 μg/l, all *p* < 0.01), after adjustment for sex, age, BMI, current smoking and current alcohol intake and the family history of diabetes. There was no statistical difference between these four groups with dysregulation of glucose metabolism (all p > 0.05) (Fig. 1).

As shown in Table 3, increased OPG was associated with a higher risk of hyperglycaemia. The higher level of OPG had a higher risk of impaired glucose regulation (OR 1.108, 95 % CI 1.009–1.117, *p* = 0.01) after adjustment for sex, age, current smoking and alcohol intake, family history of diabetes, BMI, waist/hip ratio, HOMA-IR, triacylglycerol, total cholesterol, HDL- and LDL-cholesterol; the corresponding OR for both impaired glucose regulation and type 2 diabetes was 1.121 (95 % CI 1.101–1.141, *p* = 0.0005).

**Table 3:** The risk of impaired glucose regulation and type 2 diabetes associated with a 0.1 μg/l increase in serum OPG

Model	Adjustment	IGR (n = 730)	*p* value^a^	Impaired glucose regulation and type 2 diabetes (*n* = 1,057)	*p* value^b^
Model 1	Unadjusted	1.143 (1.112–1.174)	<0.0001	1.146 (1.127–1.167)	<0.0001
Model 2	Adjusted for age, gender, current smoking, current alcohol intake and family history of diabetes	1.137 (1.108–1.166)	<0.0001	1.141 (1.115–1.168)	<0.0001
Model 3	Further adjusted for BMI and waist/hip ratio, based on Model 2	1.134 (1.104–1.165)	<0.0001	1.136 (1.107–1.166)	<0.0001
Model 4	Further adjusted for HOMA-IR, based on Model 3	1.133 (1.103–1.164)	<0.0001	1.132 (1.103–1.161)	<0.0001
Model 5	Further adjusted for serum triacylglycerol, total cholesterol, HDL- and LDL-cholesterol, based on Model 4	1.108 (1.009–1.117)	0.01	1.121 (1.101–1.141)	0.0005

Values are ORs (95 % CI)

^a^For the risk of IGR, we defined participants with normal glucose regulation as 0 (*n* = 599) and IGR as 1 (*n* = 730), excluding patients with type 2 diabetes (*n* = 327) from the analysis

^b^For the risk of combined IGR and type 2 diabetes, we defined participants with NGR as 0 (*n* = 599) and combined IGR and type 2 diabetes as 1 (*n* = 1,057)

### Serum OPG and the risk of albuminuria

**Fig. 2:**
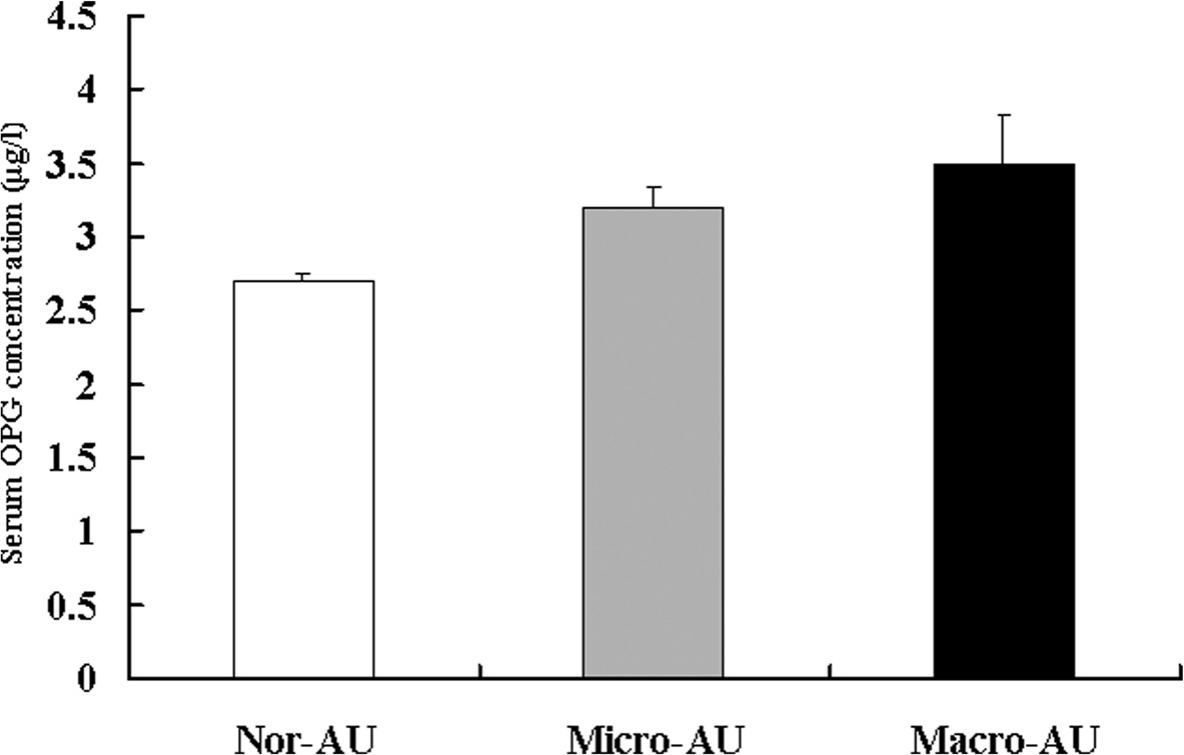
Adjusted means (±SEM) of serum OPG concentrations in participants with normal urinary albumin excretion (Nor-AU) (*n* = 1,539), microalbuminuria (Micro-AU) (*n* = 103) and macroalbuminuria (Macro-AU) (*n* = 14). The co-variables included gender, age, BMI, waist circumference, waist/hip ratio, HOMA-IR, current smoking and alcohol intake, serum triacylglycerol, plasma glucose, blood pressure, menopausal status and use of antihypertensive drugs. There was no significant difference between the groups with micro- and macroalbuminuria (*p* = 0.69), while the OPG level was significantly higher in the group with microalbuminuria than in the normal group (*p* < 0.01)

We also found that serum OPG concentrations were higher from 2.7 μg/l in participants with normal albumin excretion, to 3.2 μg/l in those with microalbuminuria, and to 3.5 μg/ml in those with macroalbuminuria (*p* < 0.01); and the difference remained statistically significant after adjustment for sex, age, BMI, waist circumference, waist/hip ratio, HOMA-IR, current smoking and alcohol intake, serum triacylglycerol, plasma glucose, blood pressure and use of antihypertensive drugs; there was no significant difference between the micro- and macroalbuminuria groups (*p* = 0.69; Fig. 2). Higher serum OPG was associated with high risk of microalbuminuria and combined micro- and macroalbuminuria (Table 4). The logistic regression analysis also showed that a 0.1 μg/l increase in OPG level was associated with an increased risk of microalbuminuria and combined micro- and macroalbuminuria of 2.5 % (*p* = 0.02) and 2.6 % (*p* = 0.005), respectively, even after adjustment for sex, age, smoking habit and alcohol intake, BMI, waist/hip ratio, HOMA-IR, estimated glomerular filtration rate, triacylglycerol, total cholesterol, and HDL- and LDL-cholesterol (Table 4).

**Table 4:** The risk of micro- and macroalbuminuria associated with a 0.1 μg/l increase in serum OPG

Model	Adjustment	Microalbuminuria (*n* = 103)	*p* value^a^	Micro- and macroalbuminuria (*n* = 117)	*p* value^b^
Model 1	Unadjusted	1.042 (1.026–1.058)	<0.0001	1.039 (1.022–1.056)	<0.0001
Model 2	Adjusted for sex, current smoking, current alcohol intake, ACEI/ARB use and family history of diabetes	1.032 (1.017–1.046)	<0.0001	1.034 (1.019–1.050)	<0.0001
Model 3	Further adjusted for BMI and WHR, based on Model 2	1.027 (1.010–1.045)	0.0007	1.029 (1.012–1.046)	<0.0001
Model 4	Further adjusted for HOMA-IR, based on Model 3	1.026 (1.010–1.042)	0.0009	1.028 (1.012–1.044)	<0.0001
Model 5	Further adjusted for GFR, based on Model 4	1.29 (1.013–1.046)	0.006	1.32 (1.018–1.046)	0.0003
Model 6	Further adjusted for lipid profiles, based on Model 5	1.025 (1.006–1.044)	0.02	1.026 (1.008–1.044)	0.005

Values are ORs (95% CI)

^a^For the risk of microalbuminuria, we defined participants with normal urinary albumin excretion as 0 (*n*=1,539) and microalbuminuria as 1 (*n*=103), excluding macroalbuminuria (*n*=14) from the logistic regression analyses

^b^For the risk of combined micro- and macroalbuminuria, we defined participants with normal urinary albumin excretion as 0 (*n*=1,539) and combined micro- and macroalbuminuria as 1 (*n*=117)

## Discussion

In this study, we found a strong association between serum OPG levels and the risk of impaired glucose regulation in a large Chinese cohort aged 40 years or older. Serum OPG concentrations were higher in the presence of isolated IFG, isolated IGT, combined IFG and IGT, and type 2 diabetes. Moreover, this association is independent of potential confounding factors. Serum OPG was also associated with a higher risk of microalbuminuria independent of various confounding factors.

Serum OPG levels were positively correlated with age, 2 h OGTT plasma glucose, HbA1c, triacylglycerol, Log_10_ ACR and blood cell count. However, in our study, there was no significant difference in the levels of OPG with respect to the duration of the disease, in contrast to the data published by other study groups [16]. Key findings in our study were that increased OPG levels increased the risk for hyperglycaemia, including impaired glucose regulation and newly diagnosed type 2 diabetes, after excluding the effects of age, gender, central obesity, HOMA-IR, family history of type 2 diabetes and the levels of serum lipid. However, given the large sample in this study, the observed association between OPG and glycemic status is relative small and of marginal statistical significance. Certainly prospective studies with solid clinical end points are urgently needed to clarify whether a high OPG level plays a causal role in the development of impaired glucose regulation.

There have also been a number of studies in healthy and obese populations revealing a negative association between OPG and insulin resistance. An inverse relationship between OPG and insulin resistance was reported in an aging male population [25] and in healthy premenopausal obese women [13]. In contrast, OPG was positively correlated with insulin resistance in an obese population and in men with type 2 diabetes mellitus. Insulin resistance could be the potential mechanism for increased serum lipid levels. We found a striking association between serum OPG and lipid levels, especially levels of triacylglycerol.

Our results also indicated that participants with a higher severity of albuminuria tended to have a higher OPG expression. We also found the independent association of OPG with risk of microalbuminuria, which coincides with the findings from previous study [26]. In the present study, the prevalence of microalbuminuria increased correspondingly and significantly with the increase of serum OPG concentration. In light of those and our results, OPG appears to be a more sensitive marker in the diabetes state and may play a vital role in diabetic nephropathy. Xiang et al [27] demonstrated that OPG was increased in microalbuminuric and macroalbuminuric type 2 diabetes mellitus patients as compared with normoalbuminuric type 2 diabetes mellitus patients. Taken together with the results of this study, these findings suggest that OPG might play an important role in diabetic nephropathy, especially when albuminuria becomes severe. OPG is produced by a variety of cell types including endothelial cells and smooth muscle cells. Therefore, the origin of the increased serum OPG levels in diabetic subjects with microvascular disease is uncertain.

In Chang’s study, which found the elevated osteoprotegerin and tumor necrosis factor related apoptosis inducing-ligand (TRAIL) concentration was associated with diabetic nephropathy. Lorz et al. explored the role of TRAIL in diabetic kidney. They demonstrated that TRAIL mRNA was upregulated in the tubulointerstitium of patients with diabetic nephropathy. The same authors observed that a high-glucose medium, characteristic of diabetes, sensitized tubular cells and podocytes to the proapoptotic effects of TRAIL suggesting that TRAIL-induced cell death could play an important role in the progression of diabetic nephropathy [28].

Type 2 diabetes and its complications were associated with wide pathophysiological pathways, including chronic low-grade systemic inflammation. We also found a significant relation between OPG and inflammation, as indicated by the levels of white blood cell counts, in our study. It has been demonstrated that OPG also positive correlated with inflammatory markers (CRP and TNF-α) [4]. These findings suggested that OPG might play a causal role in the pathogenesis of inflammation [29].

To our knowledge, this is the first study to evaluate the relationship between OPG levels and impaired glucose regulation in a large-scale population. Most potential confounders were carefully controlled, which limited the possibility of residual confounding effects. Furthermore, OPG concentrations were measured in duplicate, and the field study was completed within 2 months to minimize seasonal influences on biomarkers and other lifestyle factors. However, due to the cross-sectional nature of the present study, admittedly, we could not determine whether OPG plays a causal role in the pathogenesis of type 2 diabetes. Also, it has yet to be seen whether our results in middle-aged and older Chinese subjects can be generalized to younger populations or other ethnic groups. In the present study, we could not demonstrate any correlation between serum OPG and the presence of macrovascular disease. However, only a few patients in our study had symptoms or signs of macrovascular disease, and as our main focus was on microvascular disease, no invasive tests for macrovascular disease were performed.

## Conclusion

We have found that elevated serum OPG levels are strongly and independently associated with impaired glucose metabolism and were independently association with a higher risk of microalbuminuria in Chinese adults. Although longitudinal studies are needed, our findings provide novel insights into the potential role of OPG in the pathogenesis of type 2 diabetes as well as in the prevention and management of impaired glucose regulation.

### Ethics approval

Ethics Committee of Xinhua Hospital Affiliated to Shanghai Jiaotong University School of Medicine. The methods were carried out in accordance with the approved guidelines.

## Abbreviations

HOMA-IR: Homeostasis model assessment of insulin resistance
BMI: Body mass index
IFG: Isolated impaired fasting glucose
IGR: Impaired glucose regulation
IGT: Isolated impaired glucose tolerance
NGR: Normal glucose regulation
OPG: Osteoprotegerin
eGFR: estimated glomerular filtration rate
